# A systematic review and evidence assessment of monogenic gene-disease relationships in human male infertility

**DOI:** 10.3389/fendo.2025.1643543

**Published:** 2025-08-28

**Authors:** Qian Zhao, Huifang Peng, Jiali Chen, Hui Zhang, Yujin Ma, Hongwei Jiang

**Affiliations:** Luoyang Key Laboratory of Clinical Multiomics and Translational Medicine, Henan Key Laboratory of Rare Diseases, Endocrinology and Metabolism Center, The First Affiliated Hospital, and College of Clinical Medicine of Henan University of Science and Technology, Luoyang, China

**Keywords:** male infertility, monogenic, gene-disease relationship, next-generation sequencing, systematic review

## Abstract

**Background:**

Genetic factors play a significant role in human male infertility, with about 4% of infertile men currently identified with genetic reasons, yet most (60–70%) still lack a definitive diagnosis and remain unexplained. Similar to other medical fields, the advent of next-generation sequencing (NGS) has resulted in the discovery of a growing array of genetic variations in infertility issues affecting both genders. With the rising count of newly discovered genes, precise diagnoses are now possible for cases of male infertility that were once considered idiopathic. Nonetheless, substantial proof supporting the gene-disease relationships (GDR) remains absent in numerous instances.

**Objective and rationale:**

The year 2019 and 2021 saw the release and revision of the standardized clinical validity evaluation for monogenic reasons behind male infertility. In this report, we offer an extensive review to methodically assess all existing data (spanning from 1 Jan, 2020, to 24 Sep, 2024) regarding the singular causes of either isolated or syndromic male infertility, hormonal imbalances, or reproductive irregularities in male reproductive organs.

**Search method:**

The PRISMA protocols were utilized to gather comprehensive data from PubMed and Web of Science regarding the genetics of human male infertility and disorders of sex development (DSD) resulting in infertility, spanning from 1 January 2020 to 24 September 2024. The pathologies examined encompass both isolated infertility and syndromic male infertility, along with disorders of the endocrine and reproductive systems. A standardized scoring system was used to evaluate whether pathogenic variations in a particular gene lead to a recognized phenotype. Each GDR received a conclusive rating, ranging from no evidence to definitive.

**Outcomes:**

Out of 19885 identified and screened publications, 229 were chosen for gene and variant analysis. Our research has pinpointed 191 genes and confirmed 191 GDRs, encompassing all documented single-gene reasons for male infertility and DSD. Additionally, our research pinpointed 100 genes with at least a moderate connection to male infertility or atypical genitourinary development traits. The study did not take into account associated genetic risk factor(s) or oligogenic/polygenic causes of male infertility.

**Systematic review registration:**

http://www.crd.york.ac.uk/PROSPERO, identifier CRD42024593082.

## 1 Introduction

Infertility is a global health issue, with approximately 17.5% of the adult population (about 1 in 6 worldwide) suffering from infertility, and many people are affected by it during their lifetime (1), according to a new World Health Organization (WHO) report released today. Male infertility is the full or partial cause of infertility in 20-70% of couples (2), and normal fertility in the female partner may compensate for reduced fertility in the male partner. And low fertility in a male partner may hinder or completely eliminate conception, regardless of the fertility of the female partner. Male infertility is a complex, multifactorial pathological condition with a highly heterogeneous phenotype, ranging from complete absence of sperm in the testicles to significant changes in sperm quality, in which genetic factors play a major role (3).

Karyotype analysis (KA), azoospermia factor (AZF) microdeletion screening, and candidate gene mutation screening are part of the diagnostic examination for male infertility (4). A persistent challenge in andrological genetics lies in the lack of standardized diagnostic protocols for male infertility, where even essential evaluations—including cytogenetic karyotyping and Y chromosome microdeletion analysis (AZFa, AZFb, AZFc regions)—exhibit significant inter-laboratory variability in clinical implementation. While NGS advancements have revolutionized genetic diagnostics through high-throughput platforms enabling comprehensive whole-exome sequencing (WES) and whole-genome sequencing (WGS), this technological progress has not yet translated into consensus guidelines for systematic application in male infertility investigations. Over the past 5–10 years, the application of NGS technology in the field of male infertility has risen rapidly, helping to translate research findings into clinical practice as clinical cohorts grow. To aid this knowledge feedback, the effectiveness of screening individual genes and their relevance to certain types of infertility need to be clearly stated.

The Clinical Genome Resource (ClinGen) has established a comprehensive system for evaluating the clinical credibility of gene-disease connections (5). Another more streamlined and practical form of this model has been released recently, simplifying the evaluation of gene-disease linkages in clinical settings (6). The simplified system achieves rapid clinical decision-making by consolidating evidence categories, compressing evidence dimensions, and eliminating time validation requirements. While previous efforts, such as the comprehensive 2021 review by Houston et al., have laid the foundation for systematic evaluation of GDRs in male infertility (7), the accelerating pace of discovery and emergence of novel diagnostic tools necessitate a more updated and forward-looking synthesis. Our work aims to fulfill this unmet need by integrating recent evidence, expanding phenotype coverage. We summarized existing knowledge on male infertility and DSD between January 1, 2020 and September 24, 2024. We used the standardized clinical validity evaluation procedure of Smith et al. (6) to evaluate the clinical validity of 191 pathogenic genes involved in male infertility. This analysis allowed us to objectively classify the evidence for the involvement of genes in male infertility as non-existing, limited, moderate, strong or definitive. Findings from this study could be beneficial for research and diagnostic purposes, such as creating diagnostic gene panels, and potentially bolster genetic studies in male infertility.

## 2 Methods

### 2.1 Search strategy and study selection

The PRISMA guidelines to identify, select, appraise and synthesize studies were followed as well as for design and reporting (8). A literature search was performed as described in Brendan J. Houston et al. (7) to identify articles reporting on monogenic causes of male infertility or male reproductive system anomalies in PubMed (https://pubmed.ncbi.nlm.nih.gov/),Web of Science (WoS, https://www.webofknowledge.com),Embase (https://www.embase.com),Ovid MEDLINE (https://www.ovid.com/),Scopus (https://www.scopus.com) between 1 Jan, 2020, and 24 Sep, 2024. The search was confined to the initial research on human subjects featured in peer-reviewed English journals. The full electronic search strategy (including search terms, MeSH headings, and database-specific filters for PubMed, Web of Science, Embase, Ovid MEDLINE, Scopus) and the inclusion/exclusion criteria for screening titles, abstracts, and full texts are detailed in **Supplementary Table S1**. The relevance of titles, abstracts, and the entire text was assessed based on established criteria for inclusion and exclusion. Assessment of whether the articles met the inclusion or exclusion criteria was performed by two independent reviewers. This study, along with its associated search methodology, was recorded in the PROSPERO registry (http://www.crd.york.ac.uk/PROSPERO) under the identifier PROSPERO 2024: CRD42024593082.

### 2.2 Data selection and scoring of gene-disease relationships- extraction process

Genes and genetic variations linked to male infertility or anomalies in the male reproductive system were isolated from the chosen complete texts. Each GDR’s clinical legitimacy was assessed using a modified version of Smith et al.’s standardized scoring method (6).Two evaluators, chosen at random from a group of six authors (QZ and HFP), utilized a uniform evaluation framework to identify gene names, genetic inheritance trends, patient characteristics, discovery techniques (sequencing method), label variants, and examine both functional and clinical data. Reviewers were prohibited from rating any published GDRs to prevent partiality in assessing gene-disease and potential conflicts of interest. Under this evaluation method, each GDR received a conclusive score: no evidence (<3 points), limited (3–8 points), moderate (9–12 points), strong (13–15 points) or definitive (>15 points). Following separate evaluations, each reviewer’s individual scores (pertaining to each GDR) were analyzed, and any scoring discrepancies (>1 point variance or final classification discrepancy) were resolved by the designated reviewers. In cases where consensus was elusive, these were deliberated with all reviewers who were not in conflict. GDRs classified as moderate or above were confidently associated with human male infertility, with all study findings compiled in **Supplementary Table S2**.

## 3 Results

### 3.1 Search strategy and study selection

**Figure 1:**
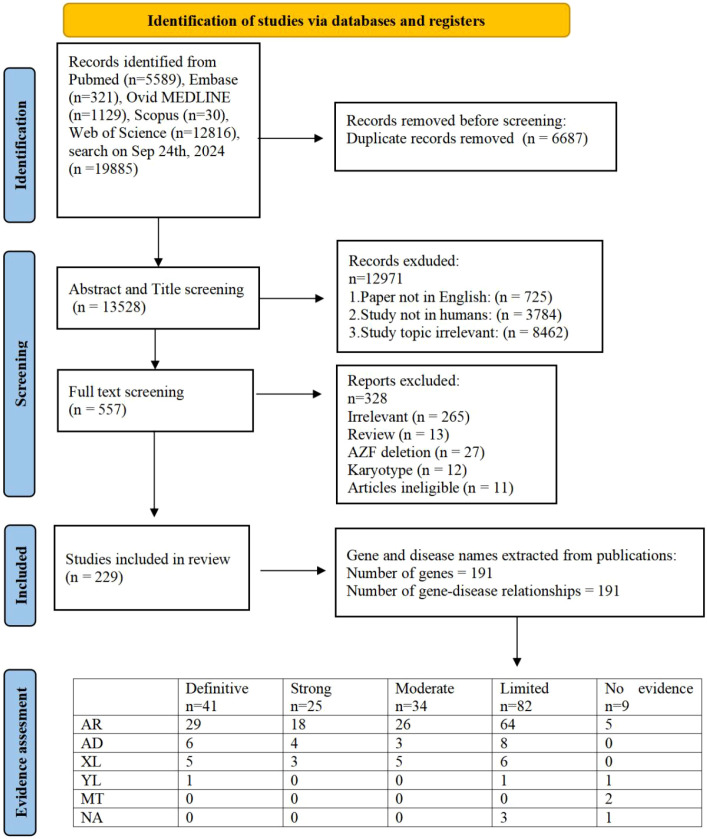
PRISMA flowchart of search and assessment process. (AR, autosomal recessive; AD, autosomal dominant; XL, X-linked; YL, Y-linked; MT, Mitochondrial Inheritance; NA, Not Available).

Our research approach was designed to uncover every publication related to the genetic aspects of male infertility, encompassing syndromes impacting the endocrine system, DSD and genitourinary anomalies. We performed a literature search using terms related to ‘male infertility’ in combination with keywords related to the word ‘genetics’ in MEDLINE-PubMed and used the same inclusion and exclusion criteria as described previously (7). We identified 20215 articles, of which 5589 were in PubMed,331 were in Embase,1439 were in Ovid MEDLINE,40 were in scopus and 12816 unique to WoS (**Figure 1**). Out of these, 557 abstracts were selected excluding publications exclusively on female infertility as well as studies on nonhuman species, pediatric cases, reviews and irrelevant topics. Out of these abstracts, 229 papers were selected for full text screening. Through a rigorous systematic review process, 229 peer-reviewed articles met the inclusion criteria for subsequent genomic data extraction. Subsequent analysis revealed 191 distinct genes of interest, which were subsequently evaluated using our novel GDR scoring system. The study selection methodology is visually summarized in **Figure 1**, which presents a PRISMA-compliant flowchart detailing the four-stage screening process: identification, screening, eligibility assessment, and final inclusion.

### 3.2 Evaluation of the gene-disease relationship

Two separate evaluators assessed the robustness and thoroughness of all data pertaining to the GDRs, employing a uniform scoring technique. The given score served to determine the clinical credibility of each GDR, which was classified into categories like no evidence, limited, moderate, strong, or definitive. The assessment included the quality of the experiment, details of patient phenotypes, gene expression-based functional data, and research on animal and cell models with *in vitro* and *in vivo* loss of function. The reclassification of variants adhered to the broadly recognized standards of the American College of Medical Genetics and Genomics-Association for Molecular Pathology (ACMG-AMP), followed by their recording in spreadsheet formats for each GDR.

The clinical validity of 191 GDRs was evaluated and 41 were classified as showing definitive evidence, 25 as strong evidence, 34 as moderate evidence, 82 as limited evidence and 9 as no evidence (**Figure 1**). Overall, 191 individual GDRs described in these 229 publications were investigated, of which 85 were newly identified and 106 were re-evaluated with our updated assessment criteria in order to incorporate any additional supporting evidence.

Of the 85 newly identified GDRs, 26 were classified as having moderate or higher evidence (**Table 1**) and 53 as having limited evidence. After classifying the existing GDRs where new evidence has been published, and using our updated scoring criteria to rate GDRs that were previously believed to be related to male infertility, 50 reports have improved since 2020 (**Supplementary Table S3**). The 56 scores were unchanged from the previous assessment, as no (or insufficient) new evidence was released during the search (**Supplementary Table S2**).

**Table 1:** Evidence quality for 26 new genes with moderate-to-definitive evidence in male infertility.

Gene	Disorder	Location	OMIM ID	Inheritance	Study count	Validation	Score in 2024	Conclusion in 2024	Change 2020 to 2024
*ACTL9*	Fertilization failure	19p13.2	619258	AR	9	4	13	Strong	New gene
*ATG4D*	Non-obstructive azoospermia	19p13.2	611340	AR	8	3	11	Moderate	New gene
*CCDC151*	Primary ciliary dyskinesia	19p13.2	616037	AR	11	6	17	Definitive	New gene
*CCDC34*	Oligoasthenoteratozoospermia	11p14.1	620084	AR	8	2	9	Moderate	New gene
*CFAP58*	Multiple morphological abnormalities of the sperm flagella	10q25.1	619144	AR	11	2	10	Moderate	New gene
*CFAP61*	Multiple morphological abnormalities of the sperm flagella	20p11.23	620409	AR	10	2	11	Moderate	New gene
*DNAAF1*	Primary ciliary dyskinesia	16q24.1	613193	AR	12	6	18	Definitive	New gene
*DNAH10*	asthenoteratozoospermia	12q24.31	619515	AR	10	3	13	Strong	New gene
*DNAH11*	Primary ciliary dyskinesia	7p15.3	611884	AR	12	6	18	Definitive	New gene
*DNAH7*	Multiple morphological abnormalities of the sperm flagella	2q32.3	620356	AR	10	2	9	Moderate	New gene
*DNAH8*	Multiple morphological abnormalities of the sperm flagella	6p21.2	619095	AR	12	6	18	Definitive	New gene
*DNHD1*	Multiple morphological abnormalities of the sperm flagella	11p15.4	619712	AR	10	2	12	Moderate	New gene
*DRC3*	asthenozoospermia	17p11.2	NA	AR	5	4	9	Moderate	New gene
*GCNA*	Non-obstructive azoospermia	Xq13.1	301077	XL	10	2	12	Moderate	New gene
*IFT140*	Oligoasthenoteratozoospermia	16p13.3	617781	AR	11	6	17	Definitive	New gene
*IGSF1*	Non-obstructive azoospermia	Xq26.1	300888	XL	10	0	8	Moderate	New gene
*LRRC23*	asthenozoospermia	12p13.31	620848	AR	5	4	9	Moderate	New gene
*MSH5*	Non-obstructive azoospermia	6p21.33	619937	AR	10	3	13	Strong	New gene
*PNLDC1*	Non-obstructive azoospermia	6q25.3	619528	AR	10	3	11	Moderate	New gene
*RSPH4A*	Primary ciliary dyskinesia	6q22.1	612649	AR	10	6	16	Definitive	New gene
*SHOC1*	Non-obstructive azoospermia	9q31.3	619949	AR	10	2	11	Moderate	New gene
*SLC26A8*	asthenoteratozoospermia	6p21.31	606766	AD	10	2	10	Moderate	New gene
*SPATA22*	Non-obstructive azoospermia	17p13.2	621001	AR	8	2	9	Moderate	New gene
*TERB1*	Non-obstructive azoospermia	16q22.1	619646	AR	8	2	9	Moderate	New gene
*TKTL1*	Non-obstructive azoospermia	Xq28	300044	XL	8	1	8	Moderate	New gene
*TTC12*	Primary ciliary dyskinesia	11q23.2	618801	AR	10	3	13	Strong	New gene

Quality of Evidence is divided into two parts: Study count and Validation. The study counts include Number of unrelated patients, Other Statistical Evidence, and Number publications reporting independent probands. Number of pathogenic variants, with a maximum total score of 12 points. Verification includes Gene Function, Gene Disruption, Model Organism, with a maximum total score of 6 points.

The combination of new and established GDR generated 100 genotypes associated with male infertility or abnormal genitourinary development phenotypes in humans, with moderate or higher evidence. Another 82 GDRs were classified as “limited” and therefore candidate genes whose dysfunction may lead to male infertility disorder (**Supplementary Table S2**). Our suggestion is that the latter group, characterized by limited evidence, will merit special attention in future years.

### 3.3 Overview of human genes involved in human male infertility

Rooted in the physiological dominance of the hypothalamic-pituitary-gonadal (HPG) axis in regulating male reproductive homeostasis, etiological classifications of infertility stratify pathology into three distinct compartments: pretesticular (endocrine dysregulation), testicular (primary gonadal failure), and post-testicular (obstructive/secretory dysfunction). Through functional annotation of genes with documented involvement in human spermatogenic impairment—supported by experimental or clinical evidence—we systematically classified these molecular candidates according to their pathophysiological contributions within this tripartite framework to delineate their mechanistic roles across the reproductive continuum.

The findings demonstrate that pre-testicular infertility predominantly manifests as syndromic conditions attributable to endocrine dysfunction, specifically exhibiting deficient sex steroid concentrations and dysregulated gonadotropin secretion. In contrast, post-testicular etiologies primarily involve anatomical obstructions that disrupt normal sperm transit from the testes. A clinically significant obstruction pattern arises from congenital vas deferens agenesis, which may present unilaterally or bilaterally (**Table 2**).

**Table 2:** Numbers of genes that are at least moderately linked to male infertility or abnormal genitourinary development phenotypes.

Description	AR	AD	XL	YL	Total
**Isolated infertility**	**49**	**5**	**8**	**1**	**63**
Acephalic sperm	3	0	0	0	3
Globozoospermia	1	0	0	0	1
Macrozoospermia	1	0	0	0	1
Multiple morphological abnormalities of the sperm flagella	14	1	0	0	15
Non-obstructive azoospermia or oligoasthenoteratozoospermia	25	4	7	1	37
Congenital bilateral absence of the vas deferens	2	0	1	0	3
Fertilization failure	3	0	0	0	3
**Syndromic infertility**	**18**	**1**	**1**	**0**	**21**
Primary ciliary dyskinesia	16	1	1	0	18
Other syndromes	3	0	0	0	3
**Endocrine disorder/Reproductive system syndrome**	**5**	**7**	**4**	**1**	**17**
Disorders of sexual development	4	2	2	1	9
Hypogonadotropic hypogonadism	1	5	2	0	8

AR, autosomal recessive; AD, autosomal dominant; XL, X-linked; YL, Y-linked.

The meanings of the bold values represent the comprehensive categories of the three types of infertility phenotypes.

The majority of confident GDRs were isolated infertility phenotypes (n=63) in 2020-2024, while a minority were linked to endocrine disorders or reproductive system syndromes (n=17) and syndromic infertility (n=21), including primary ciliary dyskinesia (PCD; **Table 2**). The majority of all genes known to cause isolated infertility phenotypes are involved in non-obstructive azoospermia (NOA) or oligoasthenoteratozoospermia (n=37, 58.73% of all 63). Patients with multiple morphological abnormalities of the sperm fagellum (MMAF) phenotypes are also of particular interest. Fifteen genes were confidently associated with MMAF in humans, 5 of which were not reported in previous studies (7). NGS can diagnose up to 50 percent of MMAF patients, although it represents only a fraction of those with isolated infertility (9). The majority of all GDRs represented an autosomal recessive inheritance pattern (n=72), while autosomal dominant (n=13), X-linked (n=13) and Y-linked (n=2) inheritance patterns were also reported.

### 3.4 Novel additions beyond previous reviews

Our study represents a significant advancement beyond the 2021 review by Houston et al., offering both temporal and methodological improvements in the evaluation of GDRs associated with male infertility. Several key novel contributions distinguish our work:

We included 229 peer-reviewed studies published between January 1, 2020, and September 24, 2024, thus expanding the evidence base beyond the time frame covered by Houston et al. (up to 2020). This allowed us to capture newly discovered genes and emerging evidence for previously reported GDRs. In addition to the databases used in prior studies (e.g., PubMed, Web of Science), we integrated additional high-impact sources such as Embase, Scopus and Ovid MEDLINE. This comprehensive search strategy increased the breadth and robustness of the included data. Among the 191 genes evaluated, 85 were newly identified since the prior review, and 26 GDRs demonstrated at least moderate clinical validity (**Table 1**). These genes, not previously included in the 2021 assessment, reflect the rapid evolution of the field and underscore the importance of continuous evidence synthesis. We re-examined 106 GDRs previously evaluated by Houston et al., applying updated scoring criteria. As a result, 50 GDRs were upgraded in their clinical validity classification based on newly available functional or clinical data. This highlights the value of dynamic reassessment as new evidence accumulates.

Our review reveals a substantial shift in diagnostic methodology, with over 81% of included studies utilizing NGS. This reflects an emerging consensus around the utility of NGS technologies and supports the feasibility of integrating genetic testing into clinical workflows. Together, these innovations provide a substantial update to the current understanding of the genetic architecture of male infertility, reinforcing the need for regular reassessments and integrative methodologies in the era of precision medicine.

## 4 Discussion

Our study builds upon the foundational work of Houston et al. (7), which systematically assessed monogenic causes of male infertility up to the year 2020. In contrast, our review expands the time frame to include all relevant studies published between January 2020 and September 2024, thereby providing a significantly updated evidence base. While we adopted a similar clinical validity scoring framework for consistency and comparability, we introduced several key methodological enhancements. For example, our review includes additional databases (e.g., Scopus and Ovid MEDLINE), We also re-evaluated previously reported gene–disease relationships under updated evidence scoring, leading to reclassification of several GDRs based on newly published functional or clinical data.

### 4.1 Clinical validity of gene–disease relationships in male infertility

In our literature search, we employed a streamlined version of the comprehensive framework used by ClinGen to curate gene-disease relationships, resulting in evidence categorized similarly to ClinGen’s method. This approach has been previously described and validated as reliable and reproducible, with outcomes that closely align with those derived from the ClinGen methodology. Consequently, it is well-suited for robust and efficient evaluation of genes in both research and diagnostic sequencing contexts (6). The results of this clinical validity assessment are dynamic and may evolve over time as the study progresses. Therefore, we anticipate that a significant number of genes currently classified as “limited” or “No evidence” may still play a crucial role in male infertility and should be valued in future genetic studies.

Importantly, Our clinical standardized assessment revealed that between January 1, 2020 and September 24, 2024, a total of 100 genes exhibited moderate-to-strong associations with male infertility or abnormal genitourinary developmental phenotypes, including 26 newly genes (**Table 1**). The 2020 report identified 104 genes and phenotypes with moderate to strong supporting evidence. Compared to the prior systematic review by Houston et al. (7), which covered gene–disease relationships from 1958 to 2020 and identified 104 genes with moderate to strong evidence, our study expands the landscape by analyzing 229 peer-reviewed articles published between 2020 and 2024. Through this, we identified 100 genes with at least moderate evidence, including 26 newly validated GDRs that had not been previously reported. Moreover, our study re-evaluated 106 previously assessed GDRs, leading to the reclassification of 50 genes whose evidence strength improved due to newly available functional or clinical data. This highlights how the increasing application of NGS and more standardized scoring systems have refined our understanding of the genetic architecture of male infertility. Unlike earlier reviews that primarily focused on isolated phenotypes such as NOA or globozoospermia, our study offers an expanded phenotype spectrum, encompassing complex syndromes such as PCD, DSD, and hormonal dysregulation syndromes. This broader scope reflects the ongoing shift from candidate-gene approaches toward comprehensive, phenotype-driven genomic assessments. Additionally, while previous reviews reported relatively few GDRs associated with sperm tail defects, our findings identified 15 genes confidently associated with MMAF, 5 of which are newly recognized (e.g., CFAP58, CFAP61, DNAH7, DNAH8, DNHD1). This further emphasizes our study’s contribution to updating the molecular basis of distinct clinical subtypes.

Evidence from animal models was often strong, and genetic studies have clearly benefited from a large number of studies of well-characterized male infertility mouse models (10). However, caution is needed when drawing conclusions about gene function and inheritance patterns based solely on mouse models. Mice and humans do not have the same reproductive systems, and genes may have (slightly) different functions or transmit disease through different patterns of inheritance (11). Statistical evidence from large humans needs to be included in the assessment to supplement evidence from animal models with datasets (12).

### 4.2 Recent advances in genetic research on male infertility (2020–2024)

In most cases, male infertility is clinically diagnosed if semen parameters are reduced. Descriptive diagnoses are “oligozoospermia” (reduced sperm count), “asthenozoospermia”(reduced sperm motility),”teratozoospermia”(reduced percentage of sperm with normal morphology). Combinations are common; most frequently “oligoasthenoteratozoospermia” or “OAT syndrome” are found. The most severe clinical phenotype is “azoospermia”, i. e. no sperm are found in the ejaculate even after centrifugation. The frequency of these phenotypes varies significantly between primary care practice and specialized centers (13). Approximately 40% of males presenting with spermatogenic dysfunction demonstrate undetermined etiology despite comprehensive diagnostic evaluation (14). Emerging evidence suggests significant genetic contributions to idiopathic cases, driving systematic exploration through diverse genomic methodologies. Initial investigations during the 1990s primarily employed targeted resequencing of genes regulating endocrine pathways, cellular metabolism/proliferation, and meiotic processes. However, subsequent validation studies yielded inconsistent replication outcomes for proposed mutations/polymorphisms, raising methodological concerns given the estimated involvement of >2,000 genes in spermatogenic regulation (15, 16). The diagnostic rate of genetic tests for all types of isolated male infertility combined currently sits between 4% and 9.2% (13, 17, 18). The reason for the low diagnosis rate of these phenotypes is mainly due to the low application of NGS methods in the field of male infertility. WES and WGS are now routinely used for diagnostic follow-up in patients with other genetic disorders, and there are currently very large genetic cohort studies (19–21).

The integration of NGS into male infertility research has catalyzed transformative discoveries between 2020 and 2024, fundamentally expanding our understanding of its genetic architecture. This review—have identified 85 novel genes associated with male infertility during this period, with 26 achieving moderate-to-definitive evidence for pathogenicity (**Table 3**). This substantial expansion of the genetic landscape, primarily driven by whole-exome/genome sequencing (adopted in 81.3% of included studies), underscores the accelerated pace of discovery enabled by high-throughput genomics.

**Table 3:** Genes associated with male infertility with moderate or higher evidence, including newly identified genes (2020-2024).

Isolated infertility									
Gene	Location	Disorder	OMIM ID	Inheritance	Score in 2020	Conclusion in 2020	Score in 2024	Conclusion in 2024	Change 2020 to 2024
ACTL7A	9q31.3	Fertilization failure	620499	AR	N/A	Unable to classify	9	Moderate	Classification increased
ACTL9*	19p13.2	Fertilization failure	619258	AR	N/A	N/A	13	Strong	New gene
ADGRG2	Xp22.13	Congenital bilateral absence of the vas deferens	300985	XL	16	Definitive	16	Definitive	No change
ARMC2	6q21	Multiple morphological abnormalities of the sperm flagella	618433	AR	11	Moderate	16	Definitive	Classification increased
ATG4D*	19p13.2	Non-obstructive azoospermia	611340	AR	N/A	N/A	11	Moderate	New gene
AURKC	19q13.43	Macrozoospermia	243060	AR	17	Definitive	17	Definitive	No change
BCORL1	Xq26.1	Oligoasthenoteratozoospermia	300688	XL	5	Limited	14	Strong	Classification increased
CCDC34*	11p14.1	Oligoasthenoteratozoospermia	620084	AR	N/A	N/A	9	Moderate	New gene
CFAP43	10q25.1	Multiple morphological abnormalities of the sperm flagella	617592	AR	17	Definitive	17	Definitive	No change
CFAP44	3q13.2	Multiple morphological abnormalities of the sperm flagella	617593	AR	17	Definitive	17	Definitive	No change
CFAP58*	10q25.1	Multiple morphological abnormalities of the sperm flagella	619144	AR	N/A	N/A	10	Moderate	New gene
CFAP61*	20p11.23	Multiple morphological abnormalities of the sperm flagella	620409	AR	N/A	N/A	12	Moderate	New gene
CFAP69	7q21.13	Multiple morphological abnormalities of the sperm flagella	617959	AR	13	Strong	13	Strong	No change
CFTR	7q31.2	Congenital bilateral absence of the vas deferens	277180	AR	17	Definitive	17	Definitive	No change
DMC1	22q13.1	Non-obstructive azoospermia	602721	AR	7	Limited	10	Moderate	Classification increased
DMRT1	9p24.3	Non-obstructive azoospermia	NA	AD	10	Moderate	14	Strong	Classification increased
DNAH1	3p21.1	Multiple morphological abnormalities of the sperm flagella	617576	AR	17	Definitive	18	Definitive	No change
DNAH10*	12q24.31	asthenoteratozoospermia	619515	AR	N/A	N/A	13	Strong	New gene
DNAH17	17q25.3	Multiple morphological abnormalities of the sperm flagella	618643	AR	15	Strong	18	Definitive	Classification increased
DNAH6	2p11.2	asthenoteratozoospermia	603336	AR	4	Limited	9	Moderate	Classification increased
DNAH7*	2q32.3	Multiple morphological abnormalities of the sperm flagella	620356	AR	N/A	N/A	12	Moderate	New gene
DNAH8*	6p21.2	Multiple morphological abnormalities of the sperm flagella	619095	AR	N/A	N/A	18	Definitive	New gene
DNHD1*	11p15.4	Multiple morphological abnormalities of the sperm flagella	619712	AR	N/A	N/A	12	Moderate	New gene
DPY19L2	12q14.2	Globozoospermia	613958	AR	16	Definitive	16	Definitive	No change
DRC3*	17p11.2	asthenozoospermia	NA	AR	N/A	N/A	9	Moderate	New gene
FANCA	16q24.3	Non-obstructive azoospermia	227650	AR	10	Moderate	18	Definitive	Classification increased
FANCM	14q21.2	azoospermia	618086	AR	13	Strong	14	Strong	No change
FSIP2	2q32.1	Multiple morphological abnormalities of the sperm flagella	618153	AR	12	Moderate	13	Strong	Classification increased
GCNA*	Xq13.1	Non-obstructive azoospermia	301077	XL	N/A	N/A	12	Moderate	New gene
IFT140*	16p13.3	Oligoasthenoteratozoospermia	617781	AR	N/A	N/A	17	Definitive	New gene
IGSF1*	Xq26.1	Non-obstructive azoospermia	300888	XL	N/A	N/A	10	Moderate	New gene
KASH5	19q13.33	Non-obstructive azoospermia	620547	AR	4	Limited	12	Moderate	Classification increased
LRRC23*	12p13.31	asthenozoospermia	620848	AR	N/A	N/A	9	Moderate	New gene
M1AP	2p13.1	Oligozoospermia	619108	AR	13	Strong	14	Strong	No change
MEIOB	16p13.3	Non-obstructive azoospermia	617706	AR	8	Limited	14	Strong	Classification increased
MSH4	1p31.1	Non-obstructive azoospermia	108420	AR	≤ 2	No evidence	10	Moderate	Classification increased
MSH5*	6p21.33	Non-obstructive azoospermia	619937	AR	N/A	N/A	13	Strong	New gene
PLCZ1	12p12.3	Fertilization failure	617214	AR	16	Definitive	16	Definitive	No change
PLK4	4q28.1	Non-obstructive azoospermia	NA	AD	7	Limited	10	Moderate	Classification increased
PMFBP1	16q22.2	Acephalic spermatozoa	618112	AR	14	Strong	15	Strong	No change
PNLDC1*	6q25.3	Non-obstructive azoospermia	619528	AR	N/A	N/A	13	Strong	New gene
QRICH2	17q25.1	Multiple morphological abnormalities of the sperm flagella	618341	AR	12	Moderate	12	Moderate	No change
RHOXF1	Xq24	Oligozoospermia	NA	XL	≤ 2	No evidence	10	Moderate	Classification increased
RXFP2	13q13.1	Non-obstructive azoospermia	NA	AR	7	Limited	11	Moderate	Classification increased
SEPTIN12	16p13.3	Multiple morphological abnormalities of the sperm flagella	614822	AD	12	Moderate	15	Strong	Classification increased
SHOC1*	9q31.3	Non-obstructive azoospermia	619949	AR	N/A	N/A	12	Moderate	New gene
SLC26A8*	6p21.31	asthenoteratozoospermia	606766	AD	N/A	N/A	12	Moderate	New gene
SLC9A3	5p15.33	Congenital bilateral absence of the vas deferens	182307	AR	4	Limited	10	Moderate	Classification increased
SPATA22*	17p13.2	Non-obstructive azoospermia	621001	AR	N/A	N/A	10	Moderate	New gene
SPEF2	5p13.2	Multiple morphological abnormalities of the sperm flagella	618751	AR	15	Strong	16	Definitive	Classification increased
STAG3	7q22.1	Non-obstructive azoospermia	619672	AR	12	Moderate	14	Strong	Classification increased
SUN5	20q11.21	Acephalic spermatozoa	617187	AR	17	Definitive	17	Definitive	No change
SYCE1	10q26.3	Non-obstructive azoospermia	616950	AR	8	Limited	10	Moderate	Classification increased
SYCP2	20q13.33	oligoasthenozoospermia	258150	AD	10	Moderate	15	Strong	Classification increased
TDRD9	14q32.33	Oligozoospermia	618110	AR	8	Limited	9	Moderate	Classification increased
TERB1*	16q22.1	Non-obstructive azoospermia	619646	AR	N/A	N/A	10	Moderate	New gene
TEX11	Xq13.1	Non-obstructive azoospermia	309120	XL	16	Definitive	16	Definitive	No change
TEX15	8p12	Fertilization failure	617960	AR	13	Strong	14	Strong	No change
TKTL1*	Xq28	Non-obstructive azoospermia	300044	XL	N/A	N/A	9	Moderate	New gene
TSGA10	2q11.2	Acephalic spermatozoa	617961	AR	10	Moderate	13	Strong	Classification increased
USP26	Xq26.2	Oligozoospermia	301101	XL	10	Moderate	10	Moderate	No change
ZMYND15	17p13.2	Oligoasthenoteratozoospermia	615842	AR	4	Limited	12	Moderate	Classification increased
Syndromic infertility									
ADCY10	1q24.2	Primary ciliary dyskinesia	143870	AD	8	Limited	11	Moderate	Classification increased
ARL2BP	16q13	Asthenozoospermia and Retinitis Pigmentosa	615434	AR	6	Limited	10	Moderate	Classification increased
ARMC4	10p12.1	Deafness-infertility syndrome	615451	AR	16	Definitive	16	Definitive	No change
CCDC103	17q21.31	Primary ciliary dyskinesia	614679	AR	6	Limited	14	Strong	Classification increased
CCDC151*	19p13.2	Primary ciliary dyskinesia	616037	AR	N/A	N/A	17	Definitive	New gene
CCDC39	3q26.33	Primary ciliary dyskinesia	613807	AR	13	Strong	17	Definitive	Classification increased
CCDC40	17q25.3	Primary ciliary dyskinesia	613808	AR	13	Strong	18	Definitive	Classification increased
CDC14A	1p21.2	Hearing impairment infertility male syndrome	608653	AR	9	Moderate	9	Moderate	No change
DNAAF1*	16q24.1	Primary ciliary dyskinesia	613193	AR	N/A	N/A	18	Definitive	New gene
DNAAF2	14q21.3	Primary ciliary dyskinesia	612518	AR	12	Moderate	12	Moderate	No change
DNAAF4	15q21.3	Primary ciliary dyskinesia	615482	AR	13	Strong	17	Definitive	Classification increased
DNAAF6	Xq22.3	Primary ciliary dyskinesia	300991	XL	15	Strong	16	Definitive	Classification increased
DNAH11*	7p15.3	Primary ciliary dyskinesia	611884	AR	N/A	N/A	18	Definitive	New gene
DNAH5	5p15.2	Primary ciliary dyskinesia	608644	AR	N/A	N/A	18	Definitive	Classification increased
DNAI1	9p13.3	Primary ciliary dyskinesia	244400	AR	8	Limited	17	Definitive	Classification increased
HYDIN	16q22.2	Primary ciliary dyskinesia	608647	AR	6	Limited	18	Definitive	Classification increased
LRRC6	8q24.22	Primary ciliary dyskinesia	614935	AR	14	Strong	15	Strong	No change
MNS1	15q21.3	Primary ciliary dyskinesia	618948	AR	10	Moderate	13	Strong	Classification increased
RSPH3	6q25.3	Primary ciliary dyskinesia	616481	AR	10	Moderate	10	Moderate	No change
RSPH4A*	6q22.1	Primary ciliary dyskinesia	612649	AR	N/A	N/A	16	Definitive	New gene
TTC12*	11q23.2	Primary ciliary dyskinesia	618801	AR	N/A	N/A	13	Strong	New gene
Endocrine disorder/Reproductive system syndrome									
AMH	19p13.3	Persistent Müllerian duct syndrome	261550	AR	17	Definitive	17	Definitive	No change
AMHR2	12q13.13	Persistent Müllerian duct syndrome	261550	AR	17	Definitive	17	Definitive	No change
ANOS1	Xp22.31	Kallmann syndrome	308700	XL	13	Strong	14	Strong	No change
AR	Xq12	46,XY Disorders of sexual development	312300	XL	17	Definitive	17	Definitive	No change
CHD7	8q12.2	Hypogonadotropic hypogonadism	612370	AD	17	Definitive	18	Definitive	No change
CYP19A1	15q21.2	Aromatase deficiency	613546	AR	16	Definitive	16	Definitive	No change
DHH	12q13.12	46,XY Disorders of sexual development	233420	AR	8	Limited	10	Moderate	Classification increased
FGFR1	8p11.23	Kallmann syndrome	147950	AD	17	Definitive	17	Definitive	No change
ANOS1	Xp22.31	Kallmann syndrome	308700	XL	13	Strong	14	Strong	No change
KISS1R	19p13.3	Hypogonadotropic hypogonadism	614837	AR	17	Definitive	17	Definitive	No change
MAP3K1	5q11.2	46,XY Disorders of sexual development	613762	AD	8	Limited	13	Strong	Classification increased
NR0B1	Xp21.2	46,XY Disorders of sexual development	300018	XL	17	Definitive	17	Definitive	No change
NR5A1	9q33.3	46,XY Disorders of sexual development	612965	AD	17	Definitive	17	Definitive	No change
PROKR2	20p12.3	Kallmann syndrome	244200	AD	17	Definitive	17	Definitive	No change
SEMA3A	7q21.11	hypogonadotropic hypogonadism	614897	AD	16	Definitive	16	Definitive	No change
SOX10	22q13.1	Hypogonadotropic hypogonadism	613266	AD	16	Definitive	16	Definitive	No change
SRY	Yp11.2	46,XX Disorders of sexual development	400045	YL	17	Definitive	17	Definitive	No change

AR, autosomal recessive; AD, autosomal dominant; XL, X-linked; YL, Y-linked.

*New genes. New genes were identified through our systematic review of literature published between January 1, 2020, and September 24, 2024. These genes were not reported in the prior comprehensive review by Houston et al. (2021) (7), which covered publications up to 2020. In our study, a total of 27 new genes were classified as having moderate or higher evidence, among which 22 were associated with isolated infertility and 5 with syndromic infertility.

Critical breakthroughs have emerged in the genetic dissection of sperm tail defects, particularly regarding MMAF. Sperm tail defects mainly manifest in the form of MMAF, characterized by artifacts in the axonemal microtubules, mitochondrial arrangement, head and neck connection, fagellar sheath abnormalities, and short, abnormally shaped, bent, or coiled fagellum (22). In our review, research during this period established 15 genes (*ARMC2, CFAP43, CFAP44, CFAP58, CFAP61, CFAP69, DNAH1, DNAH17, DNAH7, DNAH8, DNHD1, FSIP2, QRICH2, SEPTIN12, SPEF2*) with at least moderate evidence for causality. Significantly, five of these (*CFAP58, CFAP61, DNAH7, DNAH8, DNHD1*) represent newly validated associations since 2020 (**Tables 2**, **3**). NGS-based approaches now achieve diagnostic yields approaching 50% in MMAF cohorts, transforming this severe phenotype from idiopathic to genetically characterized.

PCD, alternatively termed immotile cilia syndrome, represents an autosomal recessive disorder with multisystem involvement, manifesting as chronic sino-pulmonary infections; visceral situs anomalies; and asthenozoospermia secondary to structural/functional ciliary and flagellar anomalies (23). Notably, diagnostic challenges associated with phenotypic variability and technical limitations in ciliary ultrastructural analysis likely contribute to significant underrecognition of this condition (24, 25). While spermatozoa in the majority of PCD cases demonstrate preserved morphological integrity under conventional light microscopy, pathognomonic ultrastructural anomalies discernible exclusively via transmission electron microscopy persist - including dynein arm deficiencies, microtubular disarrangements, and radial spoke absences (26, 27). Parallel advances refined the genetic basis of syndromic infertility, notably PCD and its mechanistic links to sperm immotility. Our synthesis confirms 18 genes with robust evidence for PCD-associated male infertility (*ADCY10, ARL2BP, CCDC151, CCDC39, CCDC40, CDC14A, DNAAF1, DNAAF2, DNAAF4, DNAAF6, DNAH11, DNAH5, DNAI1, HYDIN, LRRC6, MNS1, RSPH3, RSPH4A, TTC12*), highlighting shared pathways between somatic ciliary function and sperm flagellar integrity. These studies concurrently emphasized diagnostic challenges posed by phenotypic heterogeneity and the indispensable role of transmission electron microscopy in detecting ultrastructural defects invisible to conventional semen analysis.

Beyond gene discovery, this period yielded deeper mechanistic insights. Investigations into genes such as *SEPTIN12* revealed mutations causing combined sperm head-tail malformations, linking cytoskeletal regulation to pleiotropic defects (28). Robust validation efforts using animals models and expanded clinical cohorts characterized this era. These led to the reclassification of 50 previously reported GDRs, strengthening their evidence levels based on new functional data (e.g., protein studies, animal phenocopy) or larger patient datasets. A paradigm shift toward phenotype-driven genomic analyses became firmly established. The field progressively moved beyond candidate-gene approaches to embrace comprehensive WES/WGS strategies. This shift proves essential for identifying variants in genes without prior infertility associations and for detecting potential oligogenic contributions—though the latter remains a key frontier for future investigation.

Collectively, these advances underscore the dynamic nature of male infertility genetics. The identification of 82 genes with limited evidence in this review highlights substantial discovery potential. While rising diagnostic yields—particularly for severe phenotypes like MMAF and NOA—validate NGS utility, they also emphasize the urgency of developing scalable functional validation pipelines to confirm pathogenicity and decipher molecular mechanisms.

### 4.3 Recommendations for genetic testing in male infertility

Genetic testing has become increasingly important in the management and counselling of infertile males. The two main purposes of genetic testing in infertility are: to determine genetic conditions that may be inherited by offspring and to assess conditions that may affect the success of assisted reproductive technologies (29). Current diagnostic guidelines endorse three principal genetic evaluations as standard practice in male infertility assessment: KA for chromosomal architecture; Y-chromosomal microdeletion screening for AZF region integrity; and CFTR gene mutational analysis (30).

KA is a cytogenetic method using a microscope to observe chromosomes during cell division and detect abnormalities like number changes (e.g., trisomy, deletion) or structural variations (e.g., inversion, translocation) (21). It is the primary genetic test for infertile men, finding chromosomal issues in about 15% of non-obstructive azoospermia and 4% of severe oligozoospermia cases (<10×10^6^/mL sperm) (31). EAU guidelines also recommend karyotyping for couples with recurrent miscarriage, malformation, cognitive impairment, or family infertility history, regardless of sperm count (4). The main abnormality in nonobstructive azoospermia is Klinefelter syndrome and variants (47,XXY; 46,XY/47,XXY mosaics), while structural autosomal issues (translocations, inversions) are more common in oligozoospermic men (4). The Y chromosome has short (Yp) and long (Yq) arms separated by a centromere and is about 60 megabases long. Notably, 95% of its DNA is the male-specific region (MSY), a key area controlling male development and sperm production (32). Clinically important Y chromosome microdeletions (AZFa, AZFb, AZFc) are well-studied due to their many spermatogenesis genes (33). These microdeletions don’t occur in men with normal sperm counts and are rare with counts >5×10^6^/mL (34). Current EAU guidelines thus adjust testing by semen levels: microdeletion screening is optional for low sperm counts (<5×10^6^/mL) but required for no sperm or very low counts (≤1×10^6^/mL) (35). The CFTR gene on chromosome 7 spans about 250 kb and encodes a cAMP-regulated chloride channel mainly in epithelial membranes. Over 2,000 disease-causing variants exist, with effects from unnoticeable to life-threatening organ failure (36). Over 1,500 distinct variants are known, some strongly linked to congenital bilateral absence of the vas deferens (CBAVD) (37). Careful diagnosis is vital for CBAVD as it’s often missed. All men without sperm need thorough exams to check for CBAVD, especially those with low semen volume (<1.0 mL) and acidic pH (<7.0). For infertile men with CFTR variants, protocols require CFTR carrier screening and genetic counseling for female partners, since children have 50% cystic fibrosis risk if the mother is a carrier (4).

As a revolutionary genomic tool, NGS provides high-throughput analysis of substantial genomic segments with unprecedented precision and speed, while achieving significant cost-efficiency compared to traditional sequencing methodologies. Currently, NGS has three applications: targeted sequencing (TS), WES, and WGS (38). Recent advancements in genomics have provided profound insights into the genetic underpinnings of male infertility, leveraging WES and WGS to identify novel genes and variants affecting sperm development, structure, and function (39). WES has been instrumental in detecting pathogenic variants across a spectrum of conditions related to male reproductive failure. Alternatively, WGS offers a broader genomic perspective, enabling the identification of structural variants (SVs) and complex genetic rearrangements that WES might miss. Among the 229 literatures included, 197 (85.65%) used NGS tests, among which WES tests were the most widely used (87.82%). Because the genetic etiology of male infertility is very complex, involving many genes, and most of the mutations have not been reported, it is recommended to use NGS to detect multiple related genes at the same time.

The identification of 100 genes with moderate to definitive evidence provides a robust foundation for updating diagnostic gene panels for male infertility. Clinically, incorporating these genes into routine diagnostic workflows—especially in tertiary infertility centers—may increase the diagnostic yield for idiopathic cases, particularly those presenting with severe phenotypes like NOA or MMAF. For instance, genes such as CFAP43, CFAP44, and DNAH1, which are strongly associated with MMAF, can inform the decision for sperm retrieval attempts (e.g., testicular sperm extraction) and reduce the number of unnecessary surgical interventions. Similarly, identification of CFTR or ADGRG2 mutations in patients with CBAVD has direct implications for assisted reproductive technology (ART), such as intracytoplasmic sperm injection (ICSI) and preimplantation genetic testing (PGT). Furthermore, pathogenic variants in syndromic genes such as ANOS1, NR5A1, or AMH necessitate careful genetic counseling regarding inheritance risks and potential extra-gonadal manifestations. These considerations are essential when counseling couples regarding their reproductive options, including donor sperm, PGT, or prenatal diagnosis.

Taken together, the integration of these findings into clinical practice may not only enhance the diagnostic rate but also guide personalized reproductive planning, improve ART outcomes, and ensure more informed genetic counseling for affected individuals and their partners.

### 4.4 State-of-the-art perspectives: emerging trends and technologies

The advent of long-read sequencing (LRS) represents a pivotal technological breakthrough in genetic analysis, particularly in the realm of male reproductive biology. This disruptive technology has fundamentally transformed our ability to detect and characterize SVs and haplotype variants with unprecedented precision and resolution. Unlike NGS, which relies on short reads that often fail to span complex genomic regions, LRS can sequence DNA fragments ranging from kilobases to megabases in length. This capability allows for the comprehensive detection of SVs, including those in repetitive or difficult-to-sequence areas of the genome that NGS typically misses (40). Moreover, LRS can facilitate the detection of SVs that play critical roles in spermatogenesis and male fertility. For instance, the comprehensive sequencing of the Y chromosome using LRS has added over 30 million base pairs that were previously missing, including complete structures of gene families crucial for spermatogenesis like TSPY, DAZ, and RBMY (41). This advancement not only improves the diagnostic accuracy for Y-chromosome-related infertility issues but also enables the identification of subtle genetic variations that contribute to male infertility. Overall, LRS is revolutionizing the field of genetic analysis by providing a more thorough and accurate understanding of genomic variations and their impacts on clinically relevant conditions (42). Its ability to resolve complex genomic regions, phase variants with unprecedented accuracy, and detect a wide array of SVs, is making it an indispensable asset in the quest to unravel the genetic underpinnings of male infertility.

Parallel innovations in functional validation are overcoming the translatability limitations of traditional animal models. While animal models have been pivotal for functional validation, advances in human-derived *in vitro* systems—such as CRISPR-edited germ cell lines (43), organoids (44), and induced pluripotent stem cell (iPSC)-derived gametes (45)—offer human-specific mechanistic insights. These models bridge the translational gap, enabling rapid functional validation of candidate variants and assessment of therapeutic interventions.

### 4.5 Future challenges and opportunities

As emphasized in this review, the number of identified GDR and genes associated with male infertility has increased dramatically over the past few decades. Therefore, we anticipate that an increasing number of new genes will be recognized, and a significant portion of gdr that are currently rated as below moderate evidence will gain importance in their role in male infertility. Our work will greatly facilitate basic research into male infertility genes, or how they affect fertility and how to mitigate their effects. Despite significant advances in uncovering monogenic causes of male infertility, several key challenges remain unresolved, while promising opportunities are emerging in parallel. Addressing these gaps will be essential to translating genetic insights into clinical practice.

Moving forward, one key research priority will be addressing the persistent “missing heritability” in idiopathic cases, which may be driven by oligogenic or polygenic contributions, epigenetic modifications, or complex gene–environment interactions not captured by current monogenic frameworks. Integrating polygenic risk models, epigenomic profiling, and environmental exposure data into future study designs may help uncover novel etiological pathways.

Another promising direction lies in expanding research beyond severe phenotypes such as NOA and MMAF, to include milder and under-recognized forms of male infertility. This will require recruitment of diverse and underrepresented populations to ensure that genetic discoveries are globally relevant and to uncover population-specific variants.

Functional validation remains a bottleneck for translating genetic associations into clinical practice. While animal models have been invaluable, their limitations in replicating human reproductive biology highlight the need for human-based systems, such as CRISPR-edited germ cell lines, induced pluripotent stem cell-derived gametogenesis models, and advanced organoid platforms. These approaches, combined with high-resolution imaging and single-cell omics, may provide more accurate insights into gene function and pathogenic mechanisms.

From a translational perspective, building comprehensive, curated, and regularly updated gene–disease databases will be critical for harmonizing clinical genetic testing and variant interpretation. International collaborations could facilitate standardized guidelines for genetic testing in male infertility, improving diagnostic yield and enabling personalized reproductive counseling. The incorporation of multi-omics into diagnostic workflows, particularly in tertiary infertility centers, may further refine patient stratification and treatment planning.

Finally, as genetic testing becomes increasingly integrated into infertility care, ethical considerations surrounding data privacy, incidental findings, and reproductive decision-making must be proactively addressed. Establishing robust frameworks for genetic counseling and informed consent will be essential to ensure that advances in genomic medicine translate into patient-centered benefits.

In summary, the next decade will likely see the convergence of large-scale, multi-omic discovery efforts, advanced functional genomics, and clinically embedded genetic testing, moving the field closer to comprehensive, personalized, and equitable care for men with infertility.

### 4.6 Strengths and limitations

This study allowed us to assign robust and reproducible clinical validity scores to 191 GDRs. However, our work is limited to monogenic causes of male infertility and does not take into account associated genetic risk factor(s) or oligogenic/polygenic causes of male infertility. And systematic reviews inherently suffer from publication time lags, potentially missing significant studies released after September 2024. Given the field’s dynamic nature, these validity classifications represent a temporal snapshot requiring continual reassessment.

Our study was limited to literature databases and did not include specialized genetic databases such as ClinVar or OMIM. While these resources contain valuable information about gene-disease relationships and variant classifications, we focused our systematic review on peer-reviewed publications that provided both genetic and detailed phenotypic information. Future updates to this assessment would benefit from incorporating these specialized genetic databases to provide even more comprehensive coverage of established GDRs in male infertility.

## 5 Conclusion

In this updated clinical validity assessment, we evaluated a total of 191 genes with reported monogenic association to male infertility and identified 100 gene–disease relationships with at least moderate evidence for a role in male infertility. Of these 191 genes, 85 genes were not reported in previous studies and 26 gene–disease relationships with at least moderate evidence. Our results and our objective methodology and recommendations may contribute to improving genetic testing in the research or diagnosis of male infertility.

## Data Availability

The original contributions presented in the study are included in the article/**Supplementary Material**. Further inquiries can be directed to the corresponding authors.

## Supplementary material

Supplementary Data Sheet 1 (.\SR\补充表\Supplementary Table S1.docx).

Supplementary Data Sheet 2 (.\SR\补充表\Supplementary Table S2.xlsx).

Supplementary Data Sheet 3 (.\SR\补充表\Supplementary Table S3.xlsx).
