# Correction: A systematic review and evidence assessment of monogenic gene-disease relationships in human male infertility

**DOI:** 10.3389/fendo.2025.1695432

**Published:** 2025-09-12

**Authors:** Qian Zhao, Huifang Peng, Jiali Chen, Hui Zhang, Yujin Ma, Hongwei Jiang

**Affiliations:** Luoyang Key Laboratory of Clinical Multiomics and Translational Medicine, Henan Key Laboratory of Rare Diseases, Endocrinology and Metabolism Center, The First Affiliated Hospital, and College of Clinical Medicine of Henan University of Science and Technology, Luoyang, China

**Keywords:** male infertility, monogenic, gene-disease relationship, next-generation sequencing, systematic review

In the **Abstract**, the phrase “human female infertility” was incorrect in the *Search Methods* section. This has been corrected to read “human male infertility”.

The original version of this article has been updated.

