# Supplementary material for: A systematic review and evidence assessment of monogenic gene-disease relationships in human male infertility
Source: Front Endocrinol (Lausanne). 2025 Aug 28;16:1643543. doi: 10.3389/fendo.2025.1643543 (PMC12422931; doi:10.3389/fendo.2025.1643543)
Supplement: Supplementary Data Sheet 1 — (.\SR\补充表\Supplementary Table S1.docx). [file Table1.docx]

**Supplementary Table SI** Search strategy, inclusion and exclusion criteria.

| **Search term Pubmed** | (((("men"[MeSH Terms] OR ("male"[MeSH Terms] OR "male"[All Fields])) AND "2019/09/25 00:00":"3000/01/01 05:00"[Date - Publication] AND (("genetics"[MeSH Terms] OR "genetics"[MeSH Subheading] OR "genetics"[All Fields] OR "genetic"[All Fields] OR "Genetic Variation"[MeSH Terms] OR "variant"[All Fields] OR "variants"[All Fields] OR "CNV"[All Fields] OR "SNV"[All Fields] OR "SNP"[All Fields] OR "VUS"[All Fields] OR "mutation"[All Fields] OR "mutations"[All Fields] OR "polymorphism"[All Fields] OR "polymorphisms"[All Fields] OR "Karyotype"[MeSH Terms] OR "Karyotype"[All Fields] OR "chromosomes, human, y/genetics"[MeSH Terms] OR (("y chromosome"[All Fields] OR "chromosome Y"[All Fields] OR "y chromosome"[All Fields]) AND "microdeletion"[All Fields]) OR "AZFc deletion"[All Fields] OR "AZFb deletion"[All Fields] OR "AZFa deletion"[All Fields] OR "gr/gr deletion"[All Fields]) AND "2019/09/25 00:00":"3000/01/01 05:00"[Date - Publication]) AND (("infertility, male"[MeSH Terms] OR ("infertility"[All Fields] AND "male"[All Fields]) OR "male infertility"[All Fields] OR ("male"[All Fields] AND "infertility"[All Fields]) OR "infertile men"[All Fields] OR "azoospermia"[MeSH Terms] OR "azoospermia"[All Fields] OR "Sertoli Cell-Only Syndrome"[MeSH Terms] OR "Sertoli Cell-Only"[All Fields] OR "Aspermia"[All Fields] OR "oligospermia"[MeSH Terms] OR "oligospermia"[All Fields] OR "oligozoospermia"[All Fields] OR "cryptozoospermia"[All Fields] OR "hypospermatogenesis"[All Fields] OR "spermatogenic failure"[All Fields] OR "asthenozoospermia"[MeSH Terms] OR "asthenozoospermia"[All Fields] OR "Asthenospermia"[All Fields] OR "oligoasthenoteratozoospermia"[All Fields] OR "multiple morphological abnormalities of the sperm flagella"[All Fields] OR "MMAF"[All Fields] OR "Dysplasia of the fibrous sheath"[All Fields] OR "Kartagener Syndrome"[MeSH Terms] OR "Kartagener Syndrome"[All Fields] OR "Primary ciliary dyskinesia"[All Fields] OR "teratozoospermia"[MeSH Terms] OR "teratozoospermia"[All Fields] OR "teratospermia"[All Fields] OR "globozoospermia"[All Fields] OR "macrozoospermia"[All Fields] OR ("acephalic"[All Fields] AND ("spermatozoa"[MeSH Terms] OR "spermatozoa"[All Fields])) OR "Congenital bilateral aplasia of vas deferens"[Supplementary Concept] OR "Congenital bilateral aplasia of vas deferens"[All Fields] OR "Congenital bilateral absence of vas deferens"[All Fields] OR "CBAVD"[All Fields] OR "vas deferens/abnormalities"[MeSH Terms] OR "Cryptorchidism"[MeSH Terms] OR "Cryptorchidism"[All Fields] OR "Persistent Mullerian duct syndrome"[Supplementary Concept] OR "Persistent Mullerian duct syndrome"[All Fields] OR "Hypospadias"[MeSH Terms] OR "Hypospadias"[All Fields] OR "Kallmann Syndrome"[MeSH Terms] OR "Kallmann Syndrome"[All Fields] OR "Hypogonadism"[MeSH Terms] OR "Hypogonadism"[All Fields] OR "Klinefelter Syndrome"[MeSH Terms] OR "Klinefelter Syndrome"[All Fields] OR "XXY"[All Fields] OR "46, xx disorders of sex development"[MeSH Terms] OR "46,XY Disorders of Sex Development"[All Fields] OR "46,XY Disorders of Sex Development"[All Fields] OR "46,XX Disorders of Sex Development"[All Fields] OR "46,XX Disorders of Sex Development"[All Fields] OR "46,XY Disorder of Sex Development"[All Fields] OR "46,xy disorder of sex development"[All Fields] OR "46,XX Disorder of Sex Development"[All Fields] OR "46,XX Disorder of Sex Development"[All Fields] OR "Androgen-Insensitivity Syndrome"[MeSH Terms] OR "Androgen-Insensitivity Syndrome"[All Fields] OR "leydig cell hypoplasia"[Supplementary Concept] OR "receptors, lh"[MeSH Terms] OR "leydig cell hypoplasia"[All Fields] OR (("gonadal dysgenesis/genetics"[MeSH Terms] OR "gonadal dysgenesis, 46,xy"[MeSH Terms] OR "Gonadal Dysgenesis"[All Fields]) AND ("46,XY"[All Fields] OR "46,XY"[All Fields] OR "46XY"[All Fields]))) AND "2019/09/25 00:00":"3000/01/01 05:00"[Date - Publication])) NOT (("Plant Infertility"[MeSH Terms] OR "Fungi"[MeSH Terms] OR "Cattle"[MeSH Terms] OR "Swine"[MeSH Terms] OR "Intellectual Disability"[MeSH Terms] OR ("heart defects, congenital"[MeSH Terms] NOT ("Dextrocardia"[MeSH Terms] OR "Kartagener Syndrome"[MeSH Terms] OR "Noonan Syndrome"[MeSH Terms] OR "Turner Syndrome"[MeSH Terms] OR "Heterotaxy syndrome"[MeSH Terms])) OR "Leukoencephalopathies"[MeSH Terms] OR "Preimplantation Diagnosis"[MeSH Terms] OR "Prenatal Diagnosis"[MeSH Terms] OR "Leukemia"[MeSH Terms] OR "Histocompatibility Antigens Class I"[MeSH Terms] OR "Histocompatibility Antigens Class II"[MeSH Terms]) AND "2019/09/25 00:00":"3000/01/01 05:00"[Date - Publication])) AND (2020/01/01:2024/12/31[Date - Publication] AND "2019/09/25 00:00":"3000/01/01 05:00"[Date - Publication]) AND ("English"[Language] AND "2019/09/25 00:00":"3000/01/01 05:00"[Date - Publication])) AND (y_5[Filter]) |
| --- | --- |
| **Search term web of science** | TS=(men OR male) and Preprint Citation Index (Exclude – Database)  TS=(("genetics" OR "genetics" OR "genetics" OR "genetic" OR "Genetic Variation" OR "variant" OR "variants" OR "CNV" OR "SNV" OR "SNP" OR "VUS" OR "mutation" OR "mutations" OR "polymorphism" OR "polymorphisms" OR "Karyotype" OR "Karyotype" OR "Chromosomes, Human, Y/genetics" OR "Y chromosome" OR "chromosome Y" OR "Y-chromosome" AND "microdeletion" OR "AZFc deletion" OR "AZFb deletion" OR "AZFa deletion" OR "gr/gr deletion"))  TS=("infertility, male" OR "infertility" AND "male" OR "male infertility" OR "male" AND "infertility" OR "infertile men" OR "azoospermia" OR "azoospermia" OR "Sertoli Cell-Only Syndrome" OR "Sertoli Cell-Only" OR "Aspermia" OR "oligospermia" OR "oligospermia" OR "oligozoospermia" OR "cryptozoospermia" OR "hypospermatogenesis" OR "spermatogenic failure" OR "asthenozoospermia" OR "asthenozoospermia" OR "Asthenospermia" OR "oligoasthenoteratozoospermia" OR "multiple morphological abnormalities of the sperm flagella " OR "MMAF" OR "Dysplasia of the fibrous sheath" OR "Kartagener Syndrome" OR "Kartagener Syndrome" OR "Primary ciliary dyskinesia" OR "teratozoospermia" OR "teratozoospermia" OR "teratospermia" OR "globozoospermia" OR "macrozoospermia" OR "acephalic" AND "spermatozoa" OR "spermatozoa" OR "Congenital bilateral aplasia of vas deferens" OR "Congenital bilateral aplasia of vas deferens" OR "Congenital bilateral absence of vas deferens" OR "CBAVD" OR "Vas Deferens/abnormalities" OR "Cryptorchidism" OR "Cryptorchidism" OR "Persistent Mullerian duct syndrome" OR "Persistent Mullerian duct syndrome" OR "Hypospadias" OR "Hypospadias" OR "Kallmann Syndrome" OR "Kallmann Syndrome" OR "Hypogonadism" OR "Hypogonadism" OR "Klinefelter Syndrome" OR "Klinefelter Syndrome" OR "XXY" OR "46, XX Disorders of Sex Development" OR "46, XY Disorders of Sex Development" OR "46,XY Disorders of Sex Development" OR "46, XX Disorders of Sex Development" OR "46,XX Disorders of Sex Development" OR "46, XY Disorder of Sex Development" OR "46,XY Disorder of Sex Development" OR "46, XX Disorder of Sex Development" OR "46,XX Disorder of Sex Development" OR "Androgen-Insensitivity Syndrome" OR "androgen insensitivity syndrome" OR "Leydig Cell Hypoplasia" OR "Receptors, LH" OR "Leydig cell hypoplasia" OR "Gonadal Dysgenesis/genetics" OR "Gonadal Dysgenesis, 46,XY" OR "Gonadal Dysgenesis" AND "46,XY" OR "46, XY" OR "46XY") and Preprint Citation Index (Exclude – Database)  TS=("Plant Infertility" OR "Fungi" OR "Cattle" OR "Swine" OR "Intellectual Disability" OR "Heart Defects, Congenital" NOT "Dextrocardia" OR "Kartagener Syndrome" OR "Noonan Syndrome" OR "Turner Syndrome" OR "Heterotaxy syndrome" OR "Leukoencephalopathies" OR "Preimplantation Diagnosis" OR "Prenatal Diagnosis" OR "Leukemia" OR "Histocompatibility Antigens Class I" OR "Histocompatibility Antigens Class II") and Preprint Citation Index (Exclude – Database)  PY=(2020-2024) and Preprint Citation Index (Exclude – Database)  #1 AND #2 AND #3 NOT #4 AND #5 and Preprint Citation Index (Exclude – Database) and English (Languages) |
| **Search term Ovid** | 1 men.mp. or exp *Men/ or exp Male/  2 genetics.mp. or exp *Genetics/ or exp Human Genetics/ or exp Genetics, Population/ or exp Genetics, Medical/  3 infertility, male.mp. or exp *Infertility, Male/  4 exp Infertility/ or Plant Infertility.mp. or exp *Plant Infertility/  5 4 and 1 and 2 and 3  6 4 not 5  7 limit 6 to (english language and yr="2020 - 2024") |
| **Search term Embase** | 1 men.mp. or Humans/ or exp *Men/ or Male/  2 genetics.mp. or exp *Genetics/ or Genetics, Population/ or Genetics, Medical/  3 infertility, male.mp. or exp *Infertility, Male/  4 plant infertility.mp. or exp *Plant Infertility/ or Female/  5 1 and 2 and 3  6 5 not 4  7 limit 6 to (english language and yr="2020 - 2024") |
| **Search term Scopus** | ( TITLE-ABS-KEY ( men OR male OR males )  AND TITLE-ABS-KEY ( genetic* OR genomic* OR "human genetics" OR "population genetics" OR "medical genetics" )  AND TITLE-ABS-KEY ( "male infertility" OR "infertility in men" OR "male reproductive dysfunction" )  AND TITLE-ABS-KEY ( infertil* OR "reproductive disorder*" OR "fertility problem*" ) )  AND ( EXCLUDE ( SUBJAREA , "AGRI" ) OR EXCLUDE ( SUBJAREA , "VETE" ) )  AND ( PUBYEAR > 2019 AND PUBYEAR < 2025 )  AND ( LANGUAGE ( english ) ) |

| **Inclusion criteria** | 1. Publish in peer-reviewed journal 2. Studies concentrating on male infertility and/or defective genitourinary development 3. Studies conducted in human patients 4. Studies concentrating on finding a genetic cause 5. Studies presenting a monogenic form of infertility 6. Studies presenting original data |
| --- | --- |
| **Exclusion criteria title/abstract screening** | 1. Publication not in English 2. Studies investigating animals/plants/fungi/prokaryotes 3. Study topic is irrelevant and/or includes one of the following examples:  - Female infertility - Studies investigating polygenic or multifactorial forms of infertility - Sperm aneuploidies or chromosomal anomalies without mentioning constitutional chromosomal anomalies - Sperm DNA fragmentation or damage - mtDNA copy load - Variants described in population databases - Epigenetics/methylome - Sperm mRNA expression - Studies about patients with Prader scale 0-3 (included: scale 4, 5 and 6) - Syndromes characterized by severe physical or intellectual disability including CHARGE, Werner, Noonan, 4H, Opitz G/BBB, Gordon Holmes, Nijmegen breakage, Cabezas, MEHMO, Dilated Cardiomyopathy and Ataxia, Boucher-Neuhauser, Hartsfield syndrome, Aarskog-Scott, Huppke-Brendel, Brooks, Juberg-Marsidi, Warburg Micro, Prune Belly - Other syndromes: Thalassemia, Myhre, Waardenburg, Bardet-Biedl, Wilms Tumor, Alström, Complete Androgen Insensitivity syndrome, Swyer syndrome, Frasier syndrome, Woodhouse-Sakati, Cystic Fibrosis, Cushing’s syndrome, Gorlin-Goltz |
| **Exclusion criteria full text screening** | 1. Studies investigating genetic risk factors or associations without presuming a direct consequence of the variant on the gene/protein function 2. Deletion of multiple genes in AZF regions 3. Deletion or duplication of multiple genes 4. Studies presenting chromosomal aneuploidies or rearrangements without charging a (set of)affected gene(s) 5. Full text reveals study topic is irrelevant (based on exclusion criterion 3) 6. Full text not available |
